# General practitioners, attention deficit hyperactivity disorder and the risks of stimulant mismanagement

**DOI:** 10.3389/fmed.2025.1587298

**Published:** 2025-05-08

**Authors:** Waseem Jerjes, Ashley Arif, Azeem Majeed

**Affiliations:** ^1^Faculty of Medicine, Imperial College London, London, United Kingdom; ^2^Neurodiverse Advocate, London, United Kingdom

**Keywords:** ADHD, primary care, general practitioners, stimulant prescribing, clinical decision support systems, training and education, pharmacovigilance

## Introduction

The management of attention deficit hyperactivity disorder (ADHD) in England is increasingly shifting from specialist clinics to general practice, placing general practitioners (GPs) at the forefront of managing this complex condition. With ADHD diagnoses rising - global prevalence in adults is estimated at around 2.8% (1).

Recent evidence from a comprehensive systematic review and meta-analysis indicates that ADHD prevalence estimates vary significantly depending on methodology, with rates ranging from 1.6% in registry-based studies to as high as 5.0% in survey studies and 4.8% in two-stage clinical studies (2). Central European data further reveal that ADHD remains underdiagnosed compared to Western countries, with estimated prevalence rates projected to reach 1% by 2020—highlighting the need to expand diagnostic capacity across both specialist and primary care (3).

General practitioners are increasingly expected to prescribe and monitor medications, including stimulants like methylphenidate and lisdexamfetamine, which have high misuse and dependency potential and some long-term health consequences. Most GPs lack specialist training in ADHD and neuropharmacology, leading to concerns about stimulant mismanagement. A population-based study found that less than half of children and adolescents diagnosed with ADHD received pharmacological treatment, with atomoxetine being increasingly prescribed despite stimulant medications remaining the recommended first-line treatment (4).

While stimulants can significantly improve daily functioning for people with ADHD, their growing use in general practice brings complex clinical, ethical, and public health considerations to the forefront. Understanding how ADHD care has transitioned from specialist services to general practice is essential to appreciating the complexity of the issues now faced by GPs. This opinion paper explores the challenges GPs face in managing ADHD medication and assesses whether primary care is equipped for this public health challenge.

## Background

General practitioners have been increasingly made the gatekeepers of ADHD stimulant medications, a role normally meant for specialists. Though GPs are used to managing several chronic conditions, the unique risks associated with stimulant medications—those being misuse and dependency—demand special expertise in neuropharmacology and management of mental health. This shift raises ethical and clinical dilemmas, as many GPs feel unprepared for stimulant prescribing without specialist support.

Stimulant medications in adults are particularly concerning, as they are controlled substances with high abuse potential. GPs effectively need to balance symptom relief with their ethical responsibility regarding the potential for misuse. This adds to the already high workload through the need for monitoring for adverse effects, adjusting dosages, and trying to recognize signs of misuse. It is a delicate juggling act that often leaves GPs feeling unsupported and exposed to future risks in a health system offering minimal specialist guidance or structured protocols for long-term ADHD management.

The major challenges faced by GPs include a lack of specialist training in neurodevelopmental disorders and the prescribing of stimulants. In a UK pilot randomized controlled trial involving 109 general practitioners, a brief web-based ADHD awareness training significantly improved participants' knowledge and confidence scores, and reduced misconceptions, with gains sustained at two-week follow-up (5).

Treatment for ADHD needs to be highly individualized, involving titration of medications, monitoring of side effects, and management of comorbidities, such as anxiety and depression. However, most general practitioners have scant training in these domains during medical education and professional development (6). Whereas psychiatrists and neurologists have specific, intensive training in the management of ADHD, GPs are usually trained to have knowledge that is more superficial but covers a wider range of conditions. Frequent medication adjustments require precision that many GPs may not have the training to manage.

This issue is furthered by a lack of clear, accessible guidelines for the GP regarding long-term management of ADHD. The pressure of managing cases effectively with a lack of sufficient training increases the likelihood that doctors in general practice may become overwhelmed and perhaps make critical mistakes in the care of their patients. This shift in management has resulted in general practitioners increasingly being positioned as the primary prescribers of stimulant medications.

## Challenges faced by GPs

In addition to training gaps and structural limitations, GPs face professional, ethical, and legal pressures in their daily practice. A major issue in ADHD management is the blame GPs face for poor stimulant prescribing outcomes. GPs must balance the benefits of stimulants with their potential harms, often without adequate support (7). With stimulants being controlled drugs, there is always the potential for abuse, dependence, or diversion, which may leave GPs vulnerable to professional and legal consequences of practice. This concern is heightened by a lack of specialist support and clear guidelines on long-term management of ADHD. Shared care arrangements often leave GPs solely responsible once patients are deemed “stable.” However, stability in ADHD can be a bit transient, and the needs of the patients may alter very fast.

Particularly, GPs fear that in cases of a patient misusing their medication or developing dependency, they will be held responsible for failing to identify these signs early enough when they actually lack the resources or training to do so. GPs also fear retrospective blame, which can strain doctor-patient relationships. GPs may tend to be overcautious while prescribing, delay the necessary treatment, or under-prescribe stimulants due to fear of possible legal or regulatory action. This trepidation leaves the patients under-treated, thus opening up another set of risks. Without better legal protection and clearer protocols, this fear will always overhang ADHD care in primary settings and might affect the outcomes for both the patients and general practitioners themselves (8).

## Comparison to the opioid epidemic

A growing body of concern suggests that the trajectory of stimulant use may mirror past public health crises, most notably the opioid epidemic. The uncomfortably familiar trend in stimulant prescribing for ADHD echoes the early years of the opioid epidemic. Both are medical treatments that, while clinically beneficial, are fraught with considerable risks for misuse, dependency, and long-term harm. In the case of the stimulants methylphenidate and lisdexamfetamine, addictive potential is well-noted, but there remains an emphasis on only efficacy in symptom management for ADHD in the short run. Without safeguards, we risk a future public health crisis (8).

Patients often feel caught in this system too with very long waiting times for assessment in specialist NHS clinics (often 3 years or more). This concern is compounded by the pressures placed on GPs to manage these medications in complex cases. Stimulants carry a high risk of psychological dependence, especially among vulnerable populations, including adults with undiagnosed ADHD or co-occurring psychiatric conditions (8).

Beyond psychological dependence, the risks of diversion and community-level misuse are also significant. Apart from dependence, there is also a risk for diversion and misuse of stimulants, a controlled substance. Patients may begin using medications in higher doses than prescribed, or these medications can be sold to or shared with other individuals, furthering the risk for societal public health problems (9). Despite these risks, the lack of adequate monitoring and follow-up mechanisms leaves GPs managing complex patients without the systems in place to pick up early signs of misuse.

As demand for stimulant medication rises, these risks may become more deeply embedded in routine prescribing practices. As more patients seek stimulant medication—either for symptoms related to genuine ADHD or as cognitive enhancers—the health system risks normalizing long-term stimulant use (10). This is what happened with the opioid crisis: that started with an effort to treat legitimate conditions and spiraled into widespread misuse. The key to success is early recognition of potential harm. In the absence of stronger regulation, enhanced training, and better monitoring systems, the unrestrained increase in stimulant prescribing could again lead to a new wave of addiction and public health consequences that might have been avoidable.

## Proposals for change

To address these growing challenges, a series of practical and innovative strategies must be considered to support GPs and mitigate the risks associated with stimulant prescribing (11). Integrating Clinical Decision Support Systems (CDSS) into GP practices could provide real-time guidance. AI-driven CDSSs could provide GPs with specific evidence-based recommendations on ADHD management, such as dosage adjustments, potential drug interactions, and alerts for suspicious misuse. Integrating these systems into EHRs would reduce cognitive load and prescribing errors.

A complementary solution involves strengthening external monitoring and support systems. Another innovative idea is setting up regional pharmacovigilance teams focused on ADHD medication management. These would include teams of pharmacists, ADHD specialists, and GPs responsible for monitoring prescription trends and taking requisite interventions. A team-based model would ease GP burden and catch misuse early.

On the patient side, formalizing expectations and responsibilities can enhance adherence and safety.

Enhancing training in ADHD care is a pivotal step toward safer more equitable care in general practice. In addition to structural innovations, there is an imperative to develop nationally standardized, general practice-focused training programmes. This might involve modular online education through e-Learning for Healthcare or RCGP Learning, centered in core topics such as titration of stimulants, detection and treatment of side effects, detection of diversion or misuse, and treatment of common co-morbidities such as anxiety or drug use. In order to maximize clinical applicability, these modules would need to be case-based, updated periodically by specialists in ADHD, with embedded knowledge checks to assess understanding. Simulation-based prescribing workshops might also be integrated into protected learning time (PLT) sessions, allowing GPs to practice challenging ADHD consultations in a protected learning setting (Table 1).

**Table 1:** Proposed training strategies for improving ADHD management in general practice.

**Training strategy**	**Format and delivery**	**Core content areas**	**Delivered by**	**Addresses GP challenges**
Modular online training	Self-paced e-learning via eLfH or RCGP Learning or other platforms	Diagnosis in adults, titration, comorbidities, misuse risk, communication strategies	ADHD specialists, GP educators	Low confidence, limited specialist access, scalable reach
Simulation-based workshops (PLT)	In-person or virtual workshops during Protected Learning Time	Complex case scenarios, prescribing dilemmas, shared decision-making	Local GP trainers, ADHD mentors	Ethical prescribing, real-time clinical decision support
Consultant-Led shared care induction	Mandatory session prior to accepting shared care prescribing responsibility	Medication protocols, adverse effects, referral thresholds, legal accountability	Consultant psychiatrists	Inconsistent shared care handover, medicolegal risk
Regional ADHD mentorship networks	Local or ICS-level GP–specialist support groups	Case reviews, prescribing queries, email/virtual advisory services	GPwERs, ADHD nurses, psychiatrists	Isolation in complex cases, clinical governance support
VTS curriculum integration	Structured modules during GP Specialty Training	ADHD screening, neurodevelopmental care pathways, prescribing safety	Deanery educators, NHS Trust input	Under-prepared trainees, long-term workforce development
Accredited CPD certification	Postgraduate ADHD training module with formal recognition	Full-spectrum ADHD care, stimulant and non-stimulant pharmacology, deprescribing frameworks	Medical education bodies, RCGP, academic partners	Upskilling mid-career GPs, incentivising clinical special interest
Academic–clinical pilot collaborations	Joint ICS–university pilot schemes with research-informed training models	Outcome-tracked interventions, audit-informed modules, innovations in digital tools and monitoring	NHS education leads, universities, NIHR/HEE partnerships	Evaluation-driven innovation, high-quality localized evidence for implementation strategy

Local induction procedures and formal mentorship would also help to address gaps in confidence and knowledge. Shared care responsibilities undertaken by GPs might have to undergo consultant-led inductions in medication guidelines, long-term care plans, and ethics. The development of regional ADHD mentorship groups—involving GPwERs (GPs with extended roles), ADHD nurse specialists, and child or adult psychiatrists—would facilitate case-based discussions during scheduled meetings as well as asynchronous advice channels. Such arrangements would minimize clinical isolation and facilitate evidence-based prescribing in various settings. ADHD, along with other neurodevelopmental disorders, would also be included in the vocational training scheme (VTS) education to ensure that subsequent generations of GPs are adequately trained to address increasing ADHD-related demand.

To assist mid-career GPs and practice teams, accredited continuing professional development pathways might be implemented-offering optional modules in ADHD certification as part of overall mental health education portfolios. They would have the dual role of formal recognition upon completion of upskilling as well as providing an incentive to become an ADHD specialist in general practice settings. Last but not least, partnerships with higher education institutes and ICS-level educational teams would underpin research-linked training pilots to facilitate evidence development in terms of optimal formats of ADHD education in general practice. Enhancing such training infrastructure would not just enhance clinical quality but also decrease variability as well as mitigate medicolegal risk in such a high-demand clinical area.

To support GPs further, patient contracts could be considered: formal agreements with patients around responsibility in using medications for ADHD and adherence (12). Coupled with digital tools for adherence, such as smart pill dispensers or mobile applications that track medication use in real time, GPs would be well positioned to monitor this behavior rather than having to rely on patient self-report. Such technologies would immediately alert the GPs to anomalies and ensure immediate intervention.

Periodic reassessment and deprescribing strategies can also help reduce long-term risks. Similarly, a structured framework for stimulant deprescribing could be supplied, which includes periodic reassessments for the need of stimulants in the long run (13). A structured deprescribing framework would guide GPs in reducing long-term stimulant use when appropriate, minimizing dependency risks. The suggested framework would be similar to how deprescribing has been well implemented in polypharmacy amongst elderly patients and would put GPs in control of long-term medicines more frequently.

Embedding specialized professionals within GP practices can enable a broader model of care. Another significant suggestion is the embedding of behavioral health specialists within GP practices. These could be the professionals who will lead such non-pharmacological interventions as CBT and lifestyle counseling, and this can be done by clinical psychologists or ADHD nurse practitioners. This interdisciplinary model ensures GPs can focus on prescribing while specialists provide holistic care.

A national system for tracking prescribing and outcomes could enhance oversight and consistency. Setting up a national ADHD register is another plausible line of approach whereby prescription practices and patient outcomes across every level of care are under scrutiny. This would form a centralized system where GPs would access the patient's full medication history—a help in identifying potential red flags for misuse or doctor shopping. Second, the registry will provide feedback tools to GPs, enabling them to compare their prescribing practices against national benchmarks and influence change where necessary.

Exploring non-stimulant treatment options can offer safer alternatives for certain patients. Innovative pilot programs could investigate the utilization of non-stimulant first-line treatments of ADHD and provide more options to GPs where the use of stimulants is risky. Complementary programs funding, such as mindfulness-based therapies or neurofeedback, would offer another direction away from stimulant medications as a major approached treatment for ADHD, hence reducing dependency risks.

Finally, redesigning the structure of consultations may give GPs the time they need to manage ADHD effectively. Adaptive appointment models have the potential to support GPs in managing complex ADHD cases. Extended consultations or follow-up appointments staggered over time would give GPs the time to provide proper care, and not overrun their lists of patients.

In conclusion, the model of care concerning the management of ADHD in general practice presents significant challenges to GPs. Limited training, high prescribing risks, and inadequate support leave GPs in a precarious position amid an escalating public health concern. Innovative initiatives such as CDSS, regional pharmacovigilance teams, patient contracts, and non-stimulant alternatives should, therefore, be complemented with more robust monitoring systems and relevant legal protections that assist GPs in providing safe and effective ADHD care. A multidisciplinary approach is essential to prevent stimulant misuse and support GPs.
